# 2-Bromo­acetamide

**DOI:** 10.1107/S2414314624008630

**Published:** 2024-09-30

**Authors:** Anke Schwarzer, Manuel Stapf

**Affiliations:** a Institut für Organische Chemie, Technische Universität Bergakademie Freiberg, Leipziger Str. 29, 09599 Freiberg, Germany; Benemérita Universidad Autónoma de Puebla, México

**Keywords:** crystal structure, hydrogen bonding, C—H⋯Br inter­action, acetamide, carbamoyl group, carboxamide dimer

## Abstract

The title compound, C_2_H_4_BrNO, crystallizes in the monoclinic space group *P*2_1_/*c* with one mol­ecule in the asymmetric unit. The almost planar mol­ecules are organized *via* N—H⋯O hydrogen bonds into a ladder-type network, which can be characterized by the graph sets *R*^2^_2_(8) and *R*^2^_4_(8). In addition, the mol­ecules are connected by C—H⋯O and C—H⋯Br contacts.

## Abstract 2

The title compound, C_2_H_4_BrNO, crystallizes in the monoclinic space group *P*2_1_/*c* with one mol­ecule in the asymmetric unit.

## Graphical abstract

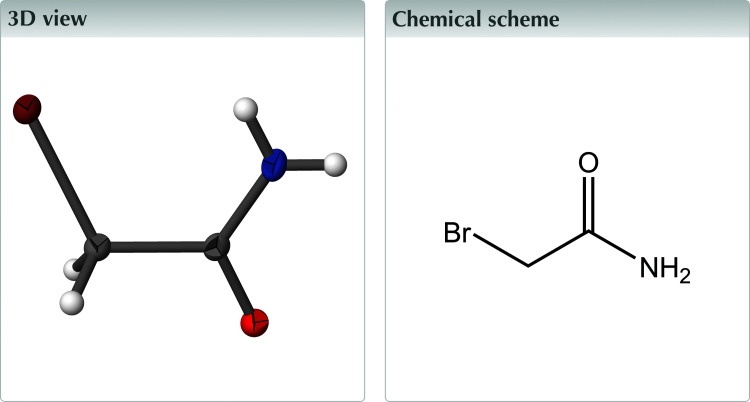

## Structure description

2-Bromo­acetamide described here is isostructural to 2-chloro­acetamide (Kalyanaraman *et al.*, 1978 ▸; Rheingold, 2021 ▸), but not to 2-fluoro­acetamide (Hughes & Small, 1962 ▸; Jeffrey *et al.*, 1981 ▸).

**Figure 1:**
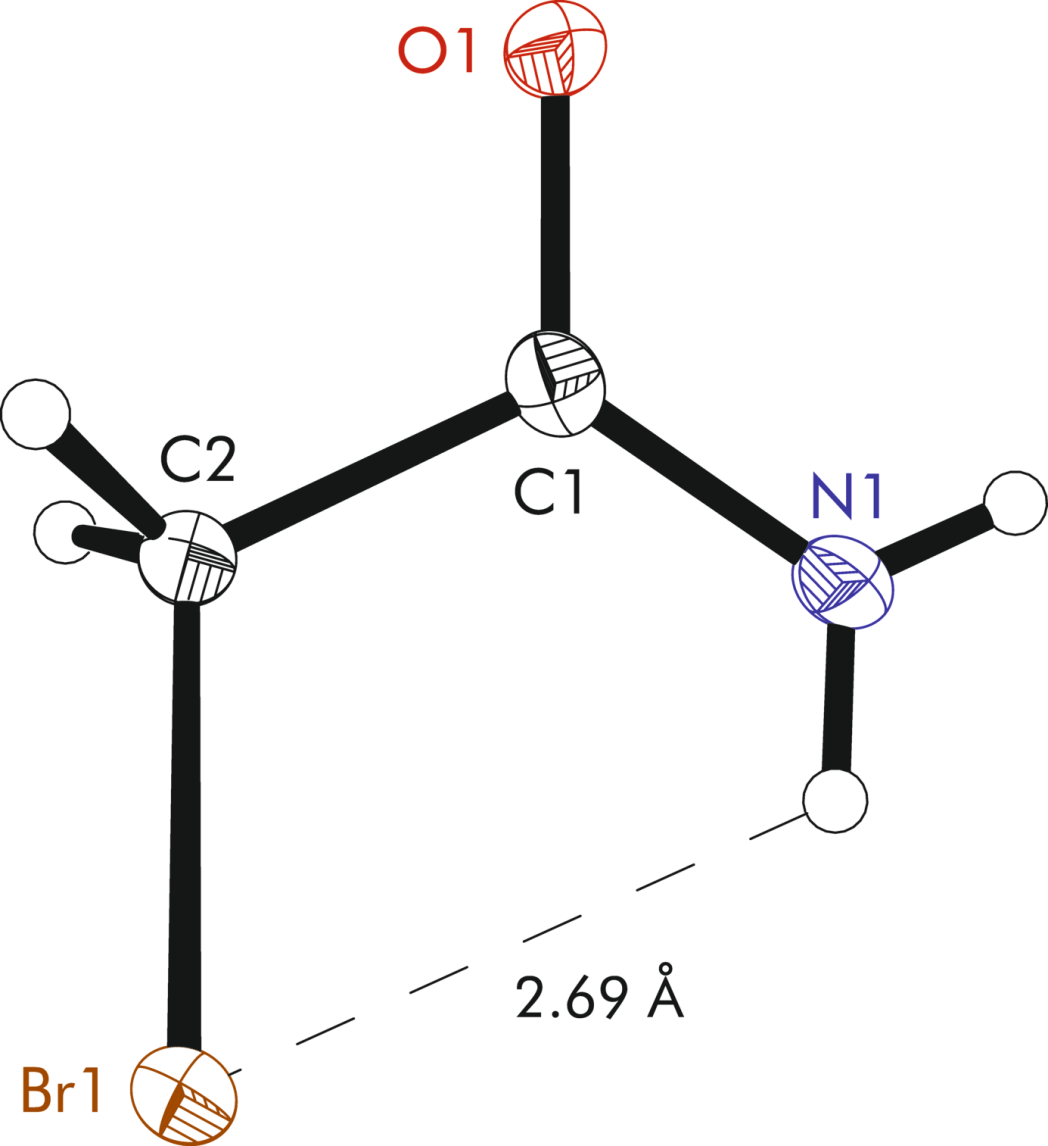
Mol­ecular structure of the title compound including the numbering scheme. Atoms are drawn with displacement ellipsoids at the 50% probability level. The intra­molecular N—H⋯Br contact is shown as dashed line.

**Figure 2:**
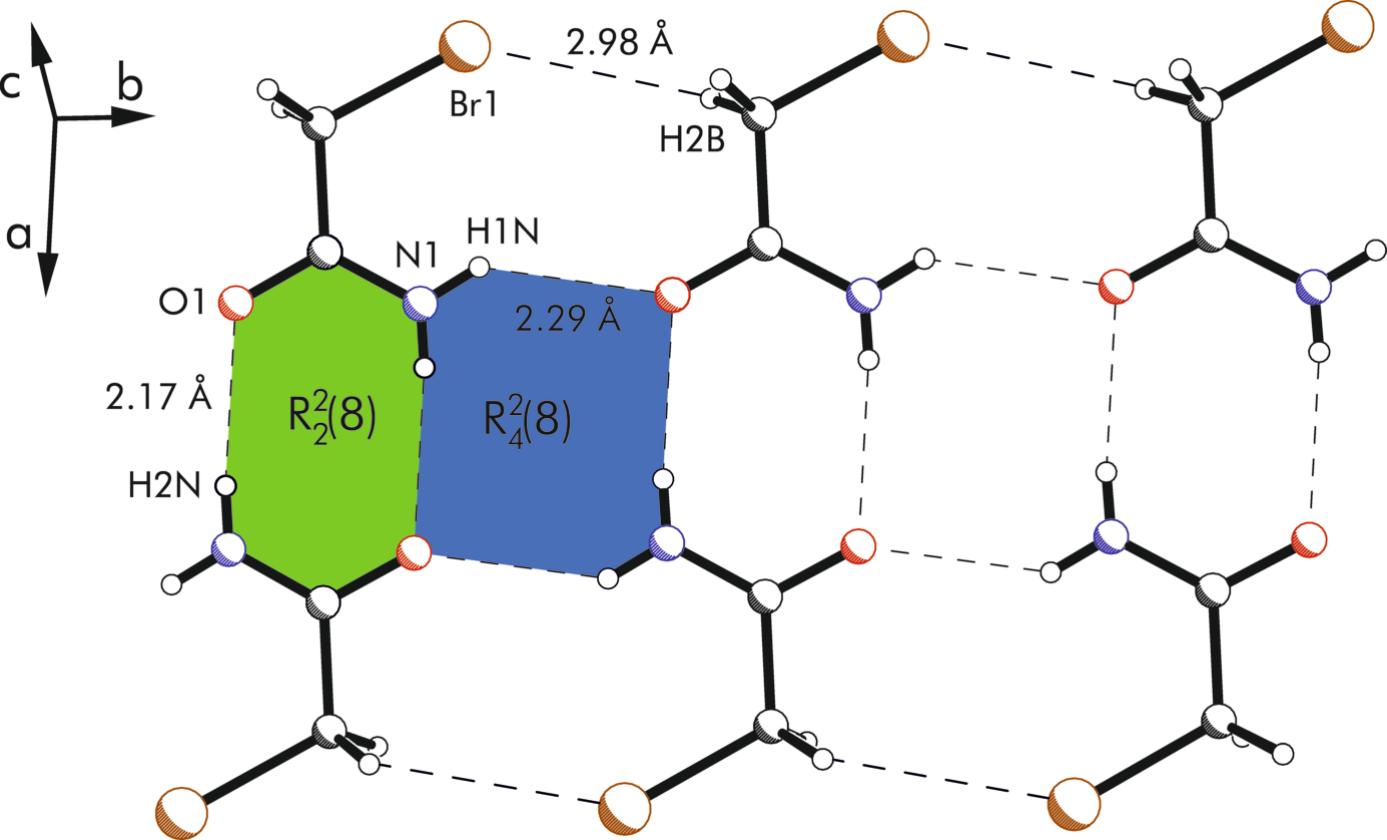
Excerpt of the crystal packing showing the 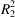
(8) and 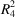
(8) motifs of the N—H⋯O=C inter­actions, as well as the C—H⋯Br contact within one layer of amide mol­ecules. These inter­actions are drawn as dashed lines.

The mol­ecular structure of the title compound is almost planar, with the bromine atom lying only slightly out of the amide plane [distance = 0.374 (3) Å] and orientated in the opposite direction to the carbonyl oxygen atom (Fig. 1 ▸). This is reflected in the torsion angles (O1/N1)—C1—C2—Br1 with values of −166.7 (2) and 14.4 (4)°, respectively. Furthermore, an intra­molecular N—H⋯Br contact of 2.69 (4) Å is observed. The crystal structure consists of layers that extend parallel to the (102) plane and are composed of centrosymmetric dimers of the title compound as the smallest supra­molecular unit (Fig. 2 ▸). Within these dimers, the mol­ecules are held together by N—H⋯O hydrogen bonds [N1—H2*N*⋯O1, 2.17 (5) Å; Table 1 ▸]. The resulting hydrogen-bonding motif, which can be described by the graph set 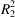
(8) (Etter, 1990 ▸, 1991 ▸; Bernstein *et al.*, 1995 ▸), is characteristic of primary carboxamides (Leiserowitz & Schmidt, 1969 ▸; Leiserowitz & Hagler, 1983 ▸; Aakeröy *et al.*, 2007 ▸) and has been observed by us previously, *e.g*., for formamide mol­ecules contained in the crystal structure of a solvate of 1-{[2,6-bis­(hy­droxy­meth­yl)-4-methyl­phen­oxy]meth­yl}-3,5-bis­{[(4,6-di­methyl­pyridin-2-yl)amino]­meth­yl}-2,4,6-tri­ethyl­benzene (Stapf *et al.*, 2023 ▸). This motif is also found for isomorphous 2-chloro­acetamide and in the structure of 2-fluoro­acetamide. As in these, the dimers in the present structure are connected by N—H⋯O bonds to form a ladder-type network. Here, the carbonyl oxygen atom additionally inter­acts with the H1*N* atom of a neighbouring mol­ecule [N1—H1*N*⋯O1, 2.29 (4) Å] and acts as a double acceptor for strong hydrogen bonds. This forms a further motif of the graph set 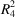
(8), as depicted in Fig. 2 ▸. The association of the dimers is supported by C—H⋯Br contacts [*d*(H⋯Br) = 2.98 Å], whereas the layers are only linked to each other *via* C—H⋯O inter­actions [*d*(H⋯O) = 2.55 Å, see Table 1 ▸].

## Synthesis and crystallization

2-Bromo­acetamide was used as purchased from Fluka. Single crystals suitable for X-ray analysis were obtained by crystallization from petroleum ether (boiling range 313–333 K) according to a literature known procedure for purification of the title compound (Halpern & Maher, 1965 ▸). ^1^H NMR (500 MHz, DMSO-*d*_6_, 298 K), δ: 3.81 (*s*, 2H, CH_2_), 7.29 (*br*, 1H, NH), 7.66 (*br*, 1H, NH) p.p.m. ^13^C NMR (125 MHz, DMSO-*d*_6_, 298 K), δ: 29.63 (CH_2_), 167.92 (C=O) p.p.m. MS (ESI): *m*/*z* calcd. for C_2_H_4_BrNONa: 159.94 [*M* + Na]^+^, found 159.94.

## Refinement

Crystal data, data collection and structure refinement details are summarized in Table 2 ▸.

## Supplementary Material

Crystal structure: contains datablock(s) I. DOI: 10.1107/S2414314624008630/bh4089sup1.cif

Structure factors: contains datablock(s) I. DOI: 10.1107/S2414314624008630/bh4089Isup2.hkl

Supporting information file. DOI: 10.1107/S2414314624008630/bh4089Isup3.cml

CCDC reference: 2381424

Additional supporting information:  crystallographic information; 3D view; checkCIF report

## Figures and Tables

**Table 1:** Hydrogen-bond geometry (Å, °)

*D*—H⋯*A*	*D*—H	H⋯*A*	*D*⋯*A*	*D*—H⋯*A*
N1—H1*N*⋯Br1	0.82 (4)	2.69 (4)	3.120 (3)	115 (3)
N1—H1*N*⋯O1^i^	0.82 (4)	2.29 (4)	2.955 (4)	140 (4)
N1—H2*N*⋯O1^ii^	0.75 (5)	2.17 (5)	2.915 (5)	172 (5)
C2—H2*B*⋯Br1^iii^	0.99	2.98	3.610 (3)	122
C2—H2*A*⋯O1^iv^	0.99	2.55	3.462 (4)	153

Symmetry codes: (i) 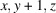; (ii) 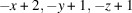; (iii) 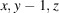; (iv) 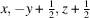.

**Table 2:** Experimental details

Crystal data	
Chemical formula	C_2_H_4_BrNO
*M*_r_	137.97
Crystal system, space group	Monoclinic, *P*2_1_/*c*
Temperature (K)	93
*a*, *b*, *c* (Å)	10.373 (4), 5.1899 (14), 7.557 (3)
β (°)	99.94 (3)
*V* (Å^3^)	400.7 (3)
*Z*	4
Radiation type	Mo *K*α
μ (mm^−1^)	10.06
Crystal size (mm)	0.11 × 0.09 × 0.04

Data collection	
Diffractometer	Stoe Stadivari
Absorption correction	Multi-scan [*X-RED32* (Stoe & Cie, 2023 ▸; Koziskova *et al.*, 2016 ▸)]
*T*_min_, *T*_max_	0.330, 0.668
No. of measured, independent and observed [*I* > 2σ(*I*)] reflections	4506, 879, 731
*R*_int_	0.036
(sin θ/λ)_max_ (Å^−1^)	0.638

Refinement	
*R*[*F*^2^ > 2σ(*F*^2^)], *wR*(*F*^2^), *S*	0.028, 0.068, 1.04
No. of reflections	879
No. of parameters	52
H-atom treatment	H atoms treated by a mixture of independent and constrained refinement
Δρ_max_, Δρ_min_ (e Å^−3^)	0.58, −0.51

Computer programs: *X-AREA* (Stoe & Cie, 2023 ▸), *SHELXT2018/2* (Sheldrick, 2015*a* ▸), *SHELXL2019/2* (Sheldrick, 2015*b* ▸), *XP* (Sheldrick, 2008 ▸), *WinGX* (Farrugia, 2012 ▸), *publCIF* (Westrip, 2010 ▸) and *ShelXle* (Hübschle *et al.*, 2011 ▸).

## full crystallographic data

### 2-Bromoacetamide Crystal data

**Table:**

C_2_H_4_BrNO	*F*(000) = 264
*M**_r_* = 137.97	*D*_x_ = 2.287 Mg m^−^^3^
Monoclinic, *P*2_1_/*c*	Mo *K*α radiation, λ = 0.71073 Å
*a* = 10.373 (4) Å	Cell parameters from 1778 reflections
*b* = 5.1899 (14) Å	θ = 1.7–22.5°
*c* = 7.557 (3) Å	µ = 10.06 mm^−^^1^
β = 99.94 (3)°	*T* = 93 K
*V* = 400.7 (3) Å^3^	Plate, colorless
*Z* = 4	0.11 × 0.09 × 0.04 mm

### 2-Bromoacetamide Data collection

**Table:**

Stoe Stadivari diffractometer	879 independent reflections
Radiation source: Primux 50 Mo	731 reflections with *I* > 2σ(*I*)
Graded multilayer mirror monochromator	*R*_int_ = 0.036
Detector resolution: 5.81 pixels mm^-1^	θ_max_ = 27.0°, θ_min_ = 2.0°
rotation method, ω scans	*h* = −13→13
Absorption correction: multi-scan [X-Red32 (Stoe & Cie, 2023; Koziskova *et al.*, 2016]	*k* = −6→6
*T*_min_ = 0.330, *T*_max_ = 0.668	*l* = −9→9
4506 measured reflections	

### 2-Bromoacetamide Refinement

**Table:**

Refinement on *F*^2^	Primary atom site location: dual
Least-squares matrix: full	Secondary atom site location: difference Fourier map
*R*[*F*^2^ > 2σ(*F*^2^)] = 0.028	Hydrogen site location: mixed
*wR*(*F*^2^) = 0.068	H atoms treated by a mixture of independent and constrained refinement
*S* = 1.04	*w* = 1/[σ^2^(*F*_o_^2^) + (0.0417*P*)^2^ + 0.0569*P*] where *P* = (*F*_o_^2^ + 2*F*_c_^2^)/3
879 reflections	(Δ/σ)_max_ = 0.001
52 parameters	Δρ_max_ = 0.58 e Å^−^^3^
0 restraints	Δρ_min_ = −0.51 e Å^−^^3^

### 2-Bromoacetamide Special details

**Table:**

Refinement. The hydrogen atoms at N1 were located in a difference Fourier map and refined with free coordinates, and with the *U*_iso_(H) values fixed at 1.5 times the equivalent *U*_eq_ value of the parent nitrogen atom (N1). The H atoms at the methylene carbon atom (C2) were included using a riding model with C—H = 0.99 Å, and the *U*_iso_(H) values were fixed at 1.2 times the equivalent *U*_eq_ value of C2.

### 2-Bromoacetamide Fractional atomic coordinates and isotropic or equivalent isotropic displacement parameters (Å^2^)

**Table:**

	*x*	*y*	*z*	*U*_iso_*/*U*_eq_	
Br1	0.61180 (3)	0.82582 (6)	0.68653 (4)	0.01566 (15)	
O1	0.8681 (3)	0.2945 (4)	0.5541 (3)	0.0181 (6)	
N1	0.8855 (3)	0.7271 (6)	0.5813 (4)	0.0174 (7)	
H1N	0.854 (4)	0.863 (7)	0.608 (6)	0.026*	
H2N	0.950 (5)	0.735 (8)	0.550 (6)	0.026*	
C1	0.8257 (3)	0.5060 (7)	0.5962 (4)	0.0140 (7)	
C2	0.6997 (3)	0.4974 (6)	0.6709 (5)	0.0160 (7)	
H2A	0.718682	0.421332	0.792727	0.019*	
H2B	0.638276	0.380045	0.594915	0.019*	

### 2-Bromoacetamide Atomic displacement parameters (Å^2^)

**Table:**

	*U*^11^	*U*^22^	*U*^33^	*U*^12^	*U*^13^	*U*^23^
Br1	0.0141 (2)	0.0146 (2)	0.0191 (2)	0.00213 (14)	0.00526 (14)	0.00064 (14)
O1	0.0169 (14)	0.0129 (12)	0.0260 (15)	0.0002 (10)	0.0078 (12)	−0.0005 (10)
N1	0.0135 (16)	0.0153 (14)	0.0251 (18)	0.0008 (13)	0.0081 (14)	−0.0024 (13)
C1	0.0114 (18)	0.0167 (16)	0.0135 (16)	0.0029 (14)	0.0013 (13)	0.0034 (14)
C2	0.0147 (19)	0.0128 (15)	0.0216 (17)	0.0012 (15)	0.0063 (14)	0.0006 (15)

### 2-Bromoacetamide Geometric parameters (Å, º)

**Table:**

Br1—C2	1.946 (3)	N1—H2N	0.75 (5)
O1—C1	1.244 (4)	C1—C2	1.512 (5)
N1—C1	1.318 (5)	C2—H2A	0.9900
N1—H1N	0.82 (4)	C2—H2B	0.9900
			
C1—N1—H1N	121 (3)	C1—C2—Br1	116.2 (2)
C1—N1—H2N	122 (3)	C1—C2—H2A	108.2
H1N—N1—H2N	117 (5)	Br1—C2—H2A	108.2
O1—C1—N1	123.7 (3)	C1—C2—H2B	108.2
O1—C1—C2	115.8 (3)	Br1—C2—H2B	108.2
N1—C1—C2	120.5 (3)	H2A—C2—H2B	107.4
			
O1—C1—C2—Br1	−166.7 (2)	N1—C1—C2—Br1	14.4 (4)

### 2-Bromoacetamide Hydrogen-bond geometry (Å, º)

**Table:**

*D*—H···*A*	*D*—H	H···*A*	*D*···*A*	*D*—H···*A*
N1—H1*N*···Br1	0.82 (4)	2.69 (4)	3.120 (3)	115 (3)
N1—H1*N*···O1^i^	0.82 (4)	2.29 (4)	2.955 (4)	140 (4)
N1—H2*N*···O1^ii^	0.75 (5)	2.17 (5)	2.915 (5)	172 (5)
C2—H2*B*···Br1^iii^	0.99	2.98	3.610 (3)	122
C2—H2*A*···O1^iv^	0.99	2.55	3.462 (4)	153

Symmetry codes: (i) *x*, *y*+1, *z*; (ii) −*x*+2, −*y*+1, −*z*+1; (iii) *x*, *y*−1, *z*; (iv) *x*, −*y*+1/2, *z*+1/2.
